# Correction to: A rare overlap of statin-induced anti-3-hydroxy-3-methyl-glutaryl-coenzyme A necrotizing autoimmune myositis and dermatomyositis

**DOI:** 10.1093/rap/rkac076

**Published:** 2022-09-10

This is a correction to: Priya Parikh, Nneoma Onuorah, Hani Kushlaf, Priyanka Vashisht, A rare overlap of statin-induced anti-3-hydroxy-3-methyl-glutaryl-coenzyme A necrotizing autoimmune myositis and dermatomyositis, *Rheumatology Advances in Practice*, Volume 5, Issue 3, 2021, rkab064, https://doi.org/10.1093/rap/rkab064

In the originally published version of this manuscript the author Hani Kushlaf was inadvertently omitted from the author list.

This error has now been corrected.

